# Chitosan-Tricarbocyanine-Based Nanogels Were Able to Cross the Blood–Brain Barrier Showing Its Potential as a Targeted Site Delivery Agent

**DOI:** 10.3390/pharmaceutics16070964

**Published:** 2024-07-21

**Authors:** Emilio Rivera López, Cecilia Samaniego López, Carla C. Spagnuolo, Bruno G. Berardino, Agustina Alaimo, Oscar E. Pérez

**Affiliations:** 1 Departamento de Química Biológica, Facultad de Ciencias Exactas y Naturales, Universidad de Buenos Aires, Ciudad Autónoma de Buenos Aires C1428EGA, Argentina; eriveralopez@qb.fcen.uba.ar (E.R.L.); bberardino@qb.fcen.uba.ar (B.G.B.); aalaimo@qb.fcen.uba.ar (A.A.); 2 Departamento de Química Orgánica, Facultad de Ciencias Exactas y Naturales, Universidad de Buenos Aires, Ciudad Autónoma de Buenos Aires C1428EGA, Argentina; cecisamaniego@qo.fcen.uba.ar (C.S.L.); carlacs@qo.fcen.uba.ar (C.C.S.); 3 Instituto de Química Biológica de la Facultad de Ciencias Exactas y Naturales, Consejo Nacional de Investigaciones Científicas y Técnicas (IQUIBICEN-CONICET), Ciudad Autónoma de Buenos Aires C1428EGA, Argentina; 4 Centro de Investigaciones en Hidratos de Carbono, Consejo Nacional de Investigaciones Científicas y Técnicas (CIHIDECAR-CONICET), Ciudad Autónoma de Buenos Aires C1428EGA, Argentina

**Keywords:** chitosan, tricarbocyanine, nanogels, SH-SY5Y cell line, blood–brain barrier

## Abstract

Targeting drugs to the central nervous system (CNS) is challenging due to the presence of the blood–brain barrier (BBB). The cutting edge in nanotechnology generates optimism to overcome the growing challenges in biomedical sciences through the effective engineering of nanogels. The primary objective of the present report was to develop and characterize a biocompatible natural chitosan (CS)-based NG that can be tracked thanks to the tricarbocyanine (CNN) fluorescent probe addition on the biopolymer backbone. FTIR shed light on the chemical groups involved in the CS and CNN interactions and between CNN-CS and tripolyphosphate, the cross-linking agent. Both in vitro and in vivo experiments were carried out to determine if CS-NGs can be utilized as therapeutic delivery vehicles directed towards the brain. An ionic gelation method was chosen to generate cationic CNN-CS-NG. DLS and TEM confirmed that these entities’ sizes fell into the nanoscale. CNN-CS-NG was found to be non-cytotoxic, as determined in the SH-SY5Y neuroblastoma cell line through biocompatibility assays. After cellular internalization, the occurrence of an endo-lysosomal escape (a crucial event for an efficient drug delivery) of CNN-CS-NG was detected. Furthermore, CNN-CS-NG administered intraperitoneally to female CF-1 mice were detected in different brain regions after 2 h of administration, using fluorescence microscopy. To conclude, the obtained findings in the present report can be useful in the field of neuro-nanomedicine when designing drug vehicles with the purpose of delivering drugs to the CNS.

## 1. Introduction

Neurological and psychiatric disorders are the main cause of poor health and disability, with a high prevalence in the world population [1,2,3]. Despite their worrying incidence, many of those disorders still do not have adequate pharmacological treatments, and both existing alternatives and those under development present certain limitations [2].

Drug delivery refers to the formulations, technologies, and systems to transport therapeutics as needed to achieve the desired effects safely and efficiently. Conventional drug delivery systems are usually accompanied by systemic side effects that are mostly attributed to their nonspecific biodistribution and uncontrollable drug-release properties [4]. Furthermore, many of the drugs have low bioavailability and cell permeability because of their low water solubility. On the other hand, the CNS is a specially protected region, and the passage of substances through it is highly regulated by the BBB [5]. Although the BBB is a natural protection system, it is an obstacle to overcome when establishing a pharmacological treatment since drugs must be administered in high doses to reach acceptable concentrations in the brain. Ergo, the latter fact results in many drugs generating unwanted effects in peripheral organs [6]. On the other hand, it is important that drugs can exert their effects in specific subcellular regions to minimize side effects and maximize their pharmacological activity. Hence, many therapeutic approaches aim to intentionally and temporarily raise BBB permeability to enhance drug delivery, as many pharmaceuticals cannot penetrate an intact BBB. In this sense, biomedical researchers are becoming more intrigued by technologies that can help them comprehend the BBB more effectively and avoid it [7].

Various methods have been explored to enhance drug delivery throughout the BBB: (i) physical methods (e.g., acoustic/mechanical stimulation, electrical stimulation, and optical/photothermal therapy) [7,8], (ii) extracellular vesicles [9], (iii) viral vectors [8], and (iv) NP [8,10]. The field of nanotechnology has made it possible to create trusted gateways across the BBB, leading to significant advances. Nanomaterials have emerged as a promising candidate to act as CNS delivery systems due to their harmless ability to cross the BBB and deliver loaded agents (genes, drugs, bioactives, etc.) [11]. In this regard, the properties of lipid-based NPs or transfersomes, polymeric, dendrimers, metals, quantum dots, and NG were studied [11,12,13,14]. Specifically, NGs comprise a 3D cross-linked network of polymers chains which can absorb considerable amounts of water or biological fluids [15]. NGs provide significant advantages over conventional drug delivery systems, which can overcome issues like poor stability and low drug-loading efficiency. NGs can be used to exert bioactive compounds or drugs’ control release, reduce adverse effects, and allow for lower therapeutic doses, thus improving patient compliance in various therapies [15,16,17,18]. In addition, NGs possess attractive properties, such as easy and low-cost production, tunability, swelling properties, biocompatibility, biodegradability, and excellent drug-loading capacity, making them ideal for biomedical applications. NGs for brain targeting are promising and can efficiently deliver therapeutic agents to the CNS by crossing the BBB [19].

CS is a polycationic amino polysaccharide that has gained a great reputation as a new functional biomaterial due to its low cost; versatility; wide availability and abundant resources; and unique properties such as antimicrobial, antioxidant, anti-inflammatory, and hemostatic activities, as well as its non-toxicity and easy functionalization [20,21]. Furthermore, its high mucoadhesion, which is an essential property for controlled and specific-site drug release, distinguishes it [20]. CS-based NGs with attractive physical and biological features are promising, effective vehicles for drug delivery, cell culture, bioimaging, and therapy [22,23]. Nowadays, CS-based NG generated via the ionic gelation method with TPP as a cross-linker is a frequently implemented technique due to its easy formulation, low toxicity, and possibility for scaling up [24]. 

NGs are too small to be directly detected by light microscopy. Thus, the main strategy employed to overcome this subject was to design fluorescent bright NGs with a detectable signal [25]. Fluorescent NGs offer unique possibilities when excited by a specific added chemical group on the biopolymer backbone, emitting light in different spectral regions. NGs can be designed combining brightness, biocompatibility, and selectivity with respect to specific tissues. Therefore, they can provide visualization of pathophysiology at scales ranging from subcellular to whole-organ levels. Due to their versatility, fluorescent NGs are attractive platforms to implement in bioimaging in biology and medicine [26].

The complexity of brain diseases and the lack of completely efficient technologies to administer drugs through the BBB constitute a true research gap for the design of new drug delivery systems. Based on the considerations detailed above, we firstly generated labelled CS with CNN, a fluorescent probe synthesized at a laboratory scale. Secondly, CNN-CS-NGs were designed and characterized by the pertinent analytical techniques. Thus, the main objective of the present report was to determine the potential capability of CNN-CS-NGs to reach the CNS and to cross the BBB. To offer a complete approach concerning the NG applications, its biocompatibility and biodistribution were studied using in vitro and in vivo models. 

## 2. Materials and Methods

### 2.1. Reagents

CS (192 kDa) was purchased in Parafarm^®^ (Saporiti S.A.C.I.F.I.A., Buenos Aires, Argentina; Ref. # 11017A). FBS, MTT, EDC, DCl, DMF, 6-aminohexanoic acid, TPP, DAPI, and Mowiol^®^ 4–88 Mw ~31000 were purchased from Sigma-Aldrich Co. (St. Louis, MO, USA). DMEM, Trypsin-EDTA 0.5% (10×), Antibiotic–Antimycotic (penicillin, streptomycin, and Amphotericin B; 100×), GlutaMaxTM-l (L-alanine-L-glutamine; 100×), Non-Essential Amino Acids (Glycine, L-alanine, L-asparagine, L-aspartic acid, L-glutamic acid, L-proline, L-serine; 100×) were from Gibco (Carlsbad, CA, USA). Other reagents were of analytical grade. Ultrapure quality water was always used.

### 2.2. Determination of Deacetylation Degree (DD, %) of CS

^1^H-NMR spectra were recorded at room temperature in DCl 5% in D_2_O as solvent, using a Bruker AM-500 NMR instrument (Bruker, Billerica, MA, USA) operating at 300 MHz for ^1^H. The ^1^H-NMR spectrum is referenced to the peak of the solvent D_2_O at δ = 4.79 ppm. The percentage of DD was calculated by using the following equation:

(1) $$\mathrm{DD}\%=\left[1-\frac{\frac{A\;({\mathrm{CH}}_{3})}{3}}{\frac{A\;(H2–H6)}{6}}\right]\times 100\;$$

where A(CH_3_) is the area of the peak corresponding to the three protons of the acetyl group (δ = 1.835 ppm), and A(H2–H6) is the sum of integral intensities of protons H2–H6 of glucosamine and N-acetyl glucosamine units.

### 2.3. Synthesis of CNN Probe and CS Labelling

The fluorescent probe was synthesized in two steps. Cl-CNN was prepared according to the previously reported procedure [27]. Briefly, CNN COOH was synthesized from CNN Cl. Cl-CNN (0.1 g, 0.13 mmol) and 6-aminohexanoic acid (0.033 g, 0.26 mmol) were dissolved in DMF (5 mL) and the solution was heated at 80 °C overnight, protected from light. The solvent was removed under vacuum, yielding an intense blue residue, which was purified by a chromatography column (Sephadex LH-20, MeOH). 

**Figure 1:**
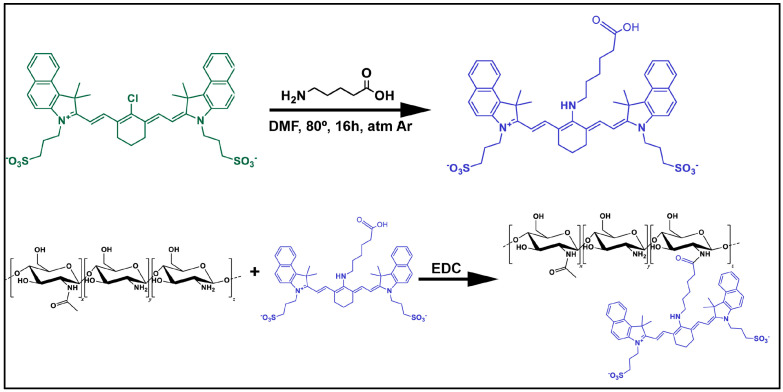
Schematic representation on the synthesis mechanism of CNN fluorescent probe (**top**) and diagram of the CNN labelling process to obtain CNN-CS biopolymer (**bottom**).

CS was covalently labelled with CNN COOH by the following procedure: a MeOH solution of CNN COOH (21.2 mg) and EDC (4.5 mg) was added dropwise to a 1% w/v solution of CS in aqueous acetic acid 1% v/v with stirring and allowed to react overnight. The reaction was dialyzed against deionized water, using a Spectra/por MW 12–14 KDa for 48 h, until no free probe was detected by fluorescence spectroscopy [23,27] (Figure 1). The quantity of CNN conjugated with CS was assessed by dissolving specified quantities of the sample in water and conducting absorbance measurements through serial dilution within the linear range, using a Cary UV spectrophotometer. The dye content in the sample was calculated based on a calibration curve previously generated for free CNN in water. The estimated obtaining was 4.3 μg dye (4.81 × 10^−3^ μmol dye) per mg conjugate.

### 2.4. Chemical Characterization of CNN Dye and CNN-CS

(a) ^1^H-NMR: spectra were recorded at room temperature, in CD3OD as solvent, using a Bruker AM-500 NMR instrument (Bruker, Billerica, MA, USA) operating at 300 MHz for ^1^H. The ^1^H-NMR spectrum is referenced to the peak of the solvent CD3OD at δ = 3.310 ppm.

(b) Absorption: The conjugate was characterized by UV-Visible spectroscopy (Cary 100, Varian, Inc., Palo Alto, CA, USA). The appearance of characteristic bands of CNN COOH is observed in CNN CS at 493, 638, and 750 nm.

(c) Mass spectrometry: LC-MS quality supplies were used to prepare the solution. The sample was analyzed by direct injection in a high-resolution mass spectrometer with electrospray ionization source and quadrupole time-of-flight analyzer model Xevo G2S Q-TOF (Waters Corp., Milford, MA, USA). The ionization source parameters were optimized to maximize the signal-to-noise ratio in ESI- and ESI+ modes.

(d) FTIR: CNN and CNN-CS samples were dried by lyophilization (The Virtis Company, Inc., Gardiner, NY, USA). Then, infrared spectra of each sample were recorded on an attenuated total reflectance FTIR-ATR Nicolet IS50 (Madison, WI, USA) (4000–400 cm^−1^; resolution 4 cm^−1^) from solids. Data were analyzed based on the average of 32 scans and 4 cm^−1^ (PerkinElmer Spectrum 100; Thermo Scientific, Waltham, MA, USA). The position and intensity of the absorption bands in the FTIR spectra were used to determine the functional groups in line with bibliography [28].

### 2.5. NG Generation

**Figure 2:**
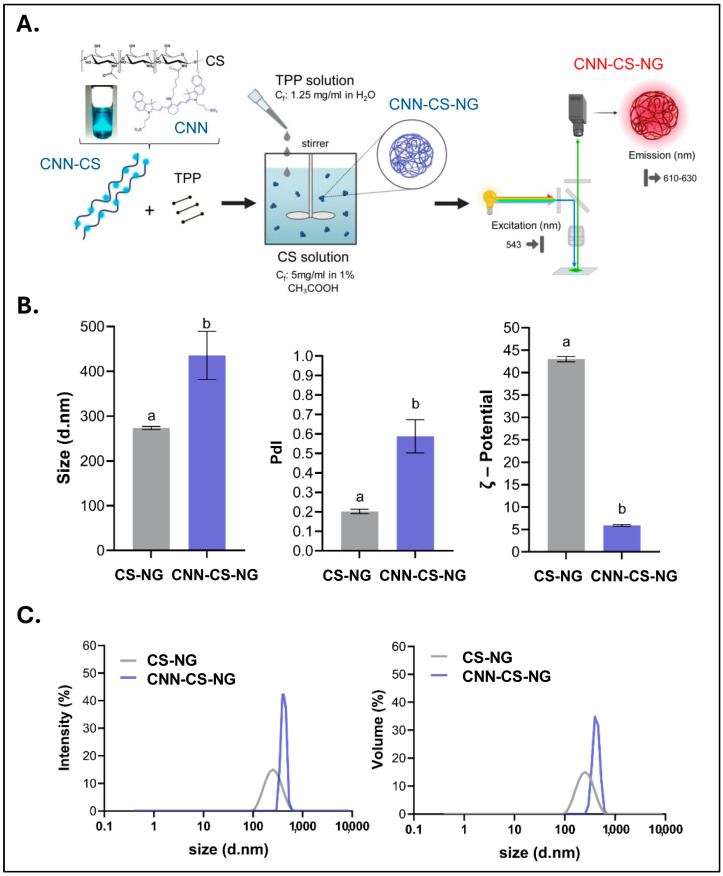
Formulation and physicochemical characterization of both CS-NG and CNN-CS-NG. (**A**) Schematic drawing of the ionic gelation method for NG generation. After light excitation at λ= 543 nm, CNN-CS-NG solutions emits at λ = 610–630 nm. (**B**) Mean particle size, polydispersity index (PdI), and ζ-Potential for both CS-NG and CNN-CS-NG. (**C**) Particle size distributions expressed in terms of intensity and volume. Different letters indicate significant differences between experimental groups (*p* < 0.01).

NGs were prepared by the ionic-gelation method [22]. Firstly, a stock solution of CS or CNN-CS (1% *w*/*v*) was dissolved in acetic acid solution (1% *v*/*v*). On the other hand, a TPP stock solution 2.5% *w*/*w* was prepared using ultra-pure water and filtered. Then, TPP was added dropwise under magnetic stirring to CS dissolution, and a colloidal suspension was instantly obtained (Figure 2A). Then, CS-NGs, which were used as control, and CNN-CS-NG suspensions were subjected to HIUS treatment for particles disaggregation under the conditions cited in Bihari et al., [29]. To this end, an ultrasonic processor (Polystat, Cole-Parmer, Vernon Hills, IL, USA) was used. Samples were HIUS-treated with a maximum net power output of 750 W at a frequency of 20 kHz, with 20% of amplitude, during 5 min, a time stablished as adequate in previous contributions [22,23]. 

### 2.6. Characterizations of CCN-NG

#### 2.6.1. Particle Size and ζ-Potential

DLS with a scattering angle of θ = 173° to the incident beam, Zetasizer Nano-ZSP, ZEN5600, Malvern Instruments (Worcestershire, UK) was employed. To obtain particle size distribution and PdI, samples were contained in disposable polystyrene cuvettes (DTS0012, Malvern Instruments, Worcestershire, UK). On the other hand, to determine the ζ-potential values, samples were placed in disposable capillary cells (DTS1060, Malvern Instruments, Worcestershire, UK). The obtained data were analyzed and interpreted according to previous reports [22,23]. 

#### 2.6.2. FTIR Analysis

Firstly, CS-NG and CNN-CS-NG samples were subjected to the protocol described in item 3.3.2, d. The position and intensity of the absorption bands in the FTIR spectra were used to determine the functional groups in line with bibliography [28].

#### 2.6.3. TEM Observation of Nanostructures

Particle morphology, distribution and shape were obtained directly by using a TEM Zeiss 109 with a Gatan W10000 camera (Carl Zeiss NTS GmbH, Oberkochen, Germany). Samples were applied onto a copper grid and air-dried for 5 min at room temperature. Then, the sample-loaded grid was negatively stained using 1% uranyl acetate for 90 s and air-dried at room temperature and processed for microscopy analysis. 

#### 2.6.4. CLSM Observation

For the visualization of CNN-CS-NG in solution, aliquots of 40 μL were placed in a 384-well glass-bottom black microplate. Fluorescence images were obtained on a Zeiss confocal LSM980 with Airyscan 2 using a 63×/1.4 objective (Zeiss, Oberkochen, Germany). The blue edition of the ZEISS ZEN 3.10 - update 3.10.1 software was used for the processing and analysis of the images obtained. The sample was excited with a λ_ex_ = 543 nm and the emission was measured at a λ_em_ = 639 nm. 

### 2.7. In Vitro Studies

#### 2.7.1. Cell Culture Conditions

Human SH-SY5Y neuroblastoma cell line (ATCC^®^ CRL-2266™) is widely used in neuroscience research as a neuronal cell model [30]. Cells were grown in DMEM supplemented with 10% heat-inactivated FBS, 2.0 mM glutamine, 1% non-essential amino acids, and 1% penicillin/streptomycin/amphotericin B. Cells were maintained in a humidified atmosphere of 5% CO_2_–95% air and 37 °C. Medium was renewed three times per week. For experiments, cells were detached with trypsin-EDTA, diluted with DMEM 10% FBS, and re-plated into multi-well plates to yield 70–80% confluent cultures after 24 h [31]. 

#### 2.7.2. Cytotoxicity Assay

Growth cells in 96-well sterile culture plates were exposed to different concentrations of NG (10, 50, 100, 500 µg/mL) for 24 h. Cell viability was evaluated by the metabolic MTT assay as previously described [22]. Absorbance was measured at λ = 570 nm with background subtraction at λ = 690 nm on a POLARstar Omega microplate reader (BMG LABTECH, Ortenberg, Germany).

#### 2.7.3. Cellular Uptake

(a) Fluorescence microscopy: cells were growth in Nunc™ 178599 Lab-Tek^®^ Chamber Slide™ System, Glass, 16-Wells. CNN-CS-NGs (50 µg/mL) were incubated in cells for 5, 15, 30, and 60 min and 24 h at culture conditions. After treatment, culture medium was removed, and cells were PBS-washed and fixed with 4% PFA. After that, cells were incubated 10 min with DAPI for nuclei staining. Confocal images were acquired in a FV1000 Olympus confocal microscope (Olympus Inc., Tokyo, Japan). Same exposure times and camera settings were fixed for imaging under the same conditions. Disparities from different slides usually occur. For that reason, a non-specific signal, as an internal control for each sample, was considered, e.g., the average intensity of background signal outside of cells. Then, from the subtracted images, fluorescent mean intensity per cell can be measured by manually sketching out the cell boundaries. Also, the normalization of each cell’s intensity was considered by multiplying the area factor. Data were rendered as the fluorescence intensity average determined by employing Fiji software, version v1.54f (NIH). In each experiment (*n* = 3), 25 cells were analyzed from at least 4 randomly chosen fields for each treatment. Digital images were optimized for contrast and brightness using Adobe Photoshop 7.0 Software.
  To quantify endo-lysosomal escape, fluorescence microscopy was used to provide information about IFP. Cells were imaged, classified, and ranked by their NG uptake, and endo-lysosomal escape efficiency was determined by using a previously described criterion [32,33]. A *punctate* fluorescence pattern is regularly considered as an indicator of the tracer compound being entrapped into vesicles; meanwhile, a *moderate* and *diffuse* cytosolic staining implies leakage from the endo-lysosomes.
(b) Flow cytometer: cells were growth in 12-well plates to be incubated then with CNN-CS-NG (50 µg/mL) for 5, 15, 30, and 60 min and 24 h at culture conditions. After treatment, cells were washed with PBS, trypsinized, resuspended in PBS and immediately subjected to flow cytometry (FACS Aria Becton Dickinson). Mean fluorescence intensity of the cell population that internalized CNN-CS-NG was determined by using FlowJo v.10.7.2 software (Tree Star Inc., Ashland, OR, USA) [34].

### 2.8. In Vivo Studies

#### 2.8.1. Animals

Female CF-1 mice (crlfcen:CF1) (ranging from 3 to 4 month of age and 30–52 g of body weight) obtained from the University of Buenos Aires Animal Facility (Bioterio Central, Facultad de Ciencias Exactas y Naturales, Argentina) were used for the in vivo experiments. Animals were housed in a room with controlled temperature (22 ± 1 °C) and humidity (50% ± 10) under filtered positive-pressure ventilation. Animals were kept in a 12:12 h light/dark cycle, with lights on at 6 am, and food and water were administered ad libitum. Experiments were performed in accordance with local regulations and the National Institutes of Health (NIH) Guide of the Care and Use of Laboratory Animals (NIH publication 80–23/96) and were previously approved by the Institutional Ethics Committee (CICUAL, Protocol No. 0024—University of Buenos Aires) [35].

#### 2.8.2. CNN-CS-NG Brain Uptake

Animals were randomly divided into three experimental groups, each group with *n* = 3, exposed according to the experimental setup [17,36]:

- Experimental groups: Mice received an intraperitoneal (i.p.) injection with 200 μL solution containing 250 µg/mL or 1000 µg/mL of CNN-CS-NG.
- Control group: Mice were injected i.p. with 200 μL of distilled water.

Mice were sacrificed 2 h after injection through cervical dislocation. Brains were immediately extracted from the skull, divided into hemispheres, and post-fixed by immersion in 4% PFA overnight at 4 °C. After that, hemispheres were transferred to PBS to cut them into 50 µm thick in a vibrating microtome (Pelco EasySlicer, Ted Pella Inc., Redding, CA, USA). Sections were stored in a cryoprotectant solution (25% glycerol, 25% ethylene glycol, 50% PBS) at −20 °C until use. Sagittal sections were obtained yielding to consecutive slices and covering most of the brain [37,38]. All slices were stained with DAPI (1 µg/mL in PBS) for 10 min, mounted, and imaged on FV1000 Olympus confocal microscope (Olympus Inc., Tokyo, Japan).

To give a quantitative measurement, fluorescent images of brain sections were submitted to an image analysis software (Fiji software, version v1.54f). Remarkably, for fluorescent quantification, images were not manipulated or over-exposed; that is to say, the potency of the laser, the excitation and emission ranges, the gain of signals, threshold limits and all other parameters were kept constant. For each treatment, the fluorescence intensity *x* area in 50 fields *per* sagittal slide was calculated. The folds increase in the mean fluorescence intensity was determined by subtracting the autofluorescence of the tissue as reference (control animals).

### 2.9. Statistical Analysis

Analyses were carried out by using GraphPad software (GraphPad Prism 8.4.3; GraphPad Software, Inc., La Jolla, CA, USA). All data are presented as means ± SEM. Furthermore, one-way analysis of variance (ANOVA) test followed by Tukey post-hoc test to compare differences between multiple groups was applied. A value of *p* < 0.05 was considered statistically significant. 

## 3. Results

### 3.1. Synthesis of the CNN Probe and Fluorescent Labelling of CS

The successful CNN synthesis and its conjugation with CS was confirmed by ^1^H-NMR (Figure S1), UV-Vis absorption and FTIR (Supplementary Figure S2), as well as with MS-ESI (M-H) (Supplementary Figure S3).

### 3.2. NG Characterization

The CS’s DD is defined as the molar fraction of D-glucosamine in the polysaccharide chain composed of both N-acetylated glucosamine and D-glucosamine. DD is a key factor since it influences the physicochemical features of the CS. There is general agreement in literature that ^1^H NMR is the best and most accurate method currently available for DD determination [39]. CS with 55–70% of DD is considered a low-grade; meanwhile, 70–85% corresponds to a medium grade. CS with 85–95% is considered of high-grade, and 95–99% of ultra-high grade. Particularly, CS with 100% of DD is difficult to produce [23]. Supplementary Figure S1 shows the ^1^H NMR spectrum that allowed us to determine that the employed CS presented a DD equal to 95.4%. Therefore, the CS used in this report corresponds to an ultra-high grade. The leitmotiv of the present report was to generate a NG that can cross the BBB. The proof of such a phenomenon will be the detection and observation of fluorescent NGin brain areas. Therefore, the physicochemical characteristics of CNN-CS-NGs formulated were first examined. 

Regarding the hydrodynamic diameter values, CS-NG was used as a control, and CNN-CS-NG exhibited a mean size centered in 273 ± 3.5 nm and 435 ± 53.8 nm, respectively. This significant increase represented around 1.55 times the average particle size because of the presence of the fluorescent probe. PdI analysis revealed variations from 0.20 ± 0.01 (NG) to 0.58 ± 0.08 (CNN-CS-NG). Towards the ζ-potential, NG presented a positive surface charge that decrease from 43.0 ± 1.2 in CS-NG to 5.9 ± 0.4 mV for CNN-CS-NG (Figure 2B). The results from DLS expressed the particle distribution size distribution in terms of intensity (%) and volume (%). Both graphs revealed a unique peak for the population analyzed: NG presented a monomodal distribution whose mean volume corresponded to the above informed (Figure 2C).

**Figure 3:**
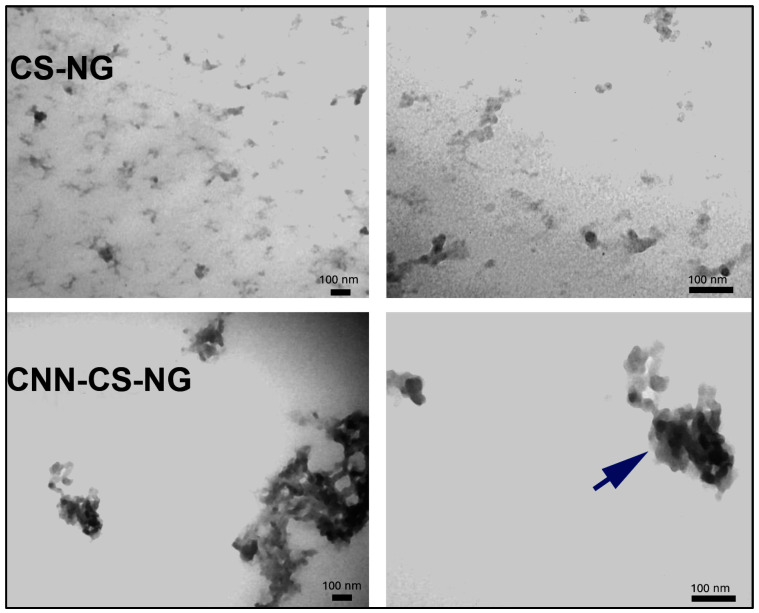
Ultrastructural characterization obtained by TEM of both CS-NG and CNN-CS-NG. Left: 50,000× magnification. Right: 85,000×. Scale bar: 100 nm. Arrow indicates CS-NG and CNN-CS-NG aggregates.

Figure 3 corresponds to TEM images revealed a spherical morphology with darker tone for both CS-NG, and CNN-CS-NG cores. There was a higher tendency to form aggregates in the system constituted by CNN-NG, which could explain the larger size of particles obtained with DLS. On the other hand, lower ζ-potential implies lower electrostatic repulsion between particles with a manifest tendency to group in clusters [40].

**Figure 4:**
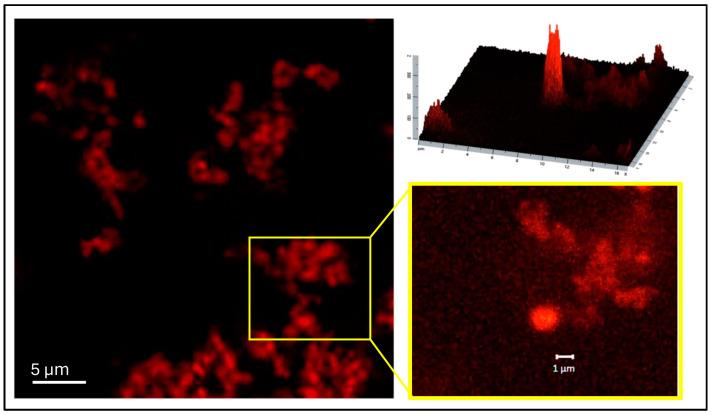
Confocal fluorescence microscopy images of CNN-CS-NG in aqueous solution (λ_ex_ = 543 nm; λ_em_ = 620 nm). Scale bar: 5 µm and 1 µm (zoom). Fluorescence profile plot (ZEISS ZEN 3.10 - update 3.10.1).

Afterwards, the CNN-CS-NG were examined in aqueous solution using confocal microscopy. In agreement with the TEM technique, CNN-NG were observed forming aggregates that resembled the FITC-CS NP generated by Khattab et al. [31] (Figure 4).

**Figure 5:**
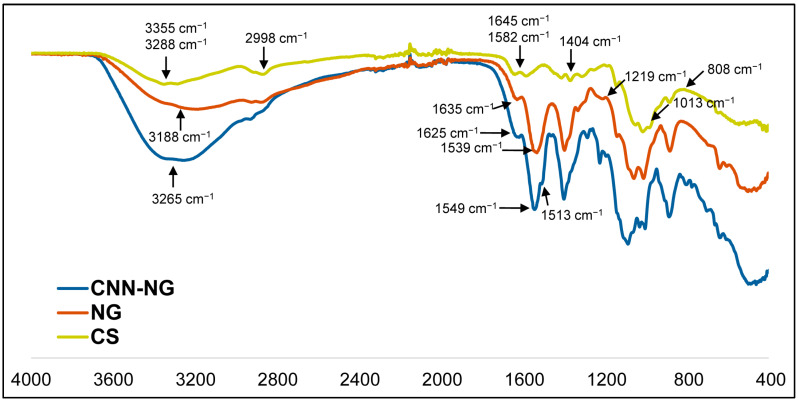
FTIR spectra for free CS molecule (yellow line), CS-NG (orange line), and CNN-CS-NG (blue line).

Figure 5 corresponds to the FTIR spectra obtained for free CS, NG, and CNN-NG. To organize the analysis, two comparisons were made: CS versus CS-NG and CS-NG versus CNN-CS-NG [41]. Progressing with the sample’s characterization, FTIR spectra were obtained for free CS, NG, and CNN-CS-NG. To organize the analysis, two comparisons were made: CS versus CS-NG and CS-NG versus CNN-CS-NG. Thus, CS macromolecule shows its characteristic peaks at 3355 and 3288 cm^−1^, typical of the O-H and N-H bonds stretching. The 2998 cm^−1^ measurement corresponds to a characteristic peak at C-H stretching. At 1645 cm^−1^, there was a typical peak of the C=O stretching of the amide II group. At 1582 cm^−1^, there was a specific peak of the vibration of an amine I (N-H bending of primary amine). At 1404 cm^−1^, there was a characteristic peak of the C-H and O-H deformation in amide II. Later, at 1013 cm^−1^ appeared the corresponding peak of the C-O/C-H stretching [22]. In the NG spectrum, a peak at 3188 cm^−1^ was seen, a region where CS showed two peaks, indicating vibrational changes of the amino (N-H) and/or hydroxyl (O-H) group. Then, the peaks seen in the CS at 1645 and 1582 cm^−1^, corresponding to the stretching of C-O (amide II) and N-H (amine I) bond, respectively, were shifted to 1635 and 1539 cm^−1^. These changes have been described as proving the electrostatic interaction between CS and TPP molecules [42]. This functional group in principle would not be directly involved in the interaction. Finally, in the spectrum of the CS-NG, a peak is observed at 1219 cm^−1^, which was not detected in the spectrum of the free CS macromolecule belongs to the presence of the P-O and/or P=O bond of the TPP’s phosphate group [22]. Then, in the CNN-CS-NG sample, the peak of 3188 cm^−1^ presented in the CS-NG, shifts to 3265 cm^−1^. This indicated vibrational changes in the amino (N-H) and/or hydroxyl (O-H) group. The peak at 1635 cm^−1^ in the CS-NG sample varies in those fluorescent NG (1625 cm^−1^). Discrepancies could be explained by the covalent binding of CNN with the CS macromolecule since the union presumably formed between the biopolymer and the CNN converts the amine I of the deacetylated unit into an amide II group. Two peaks appeared in the CNN-CS-NG spectrum, one corresponding to the amide formed in the covalent bond between the CNN and CS, and another typical of the N-acetyl-glucosamine unit. So, the peak at 1539 cm^−1^ for the CS-NG, which corresponds to the amide group changes in CNN-CS-NG sample (1513 and 1549 cm^−1^).

### 3.3. Biological Performance of CNN-CS-NG: Cellular Biocompatibility, Uptake, and Imaging

**Figure 6:**
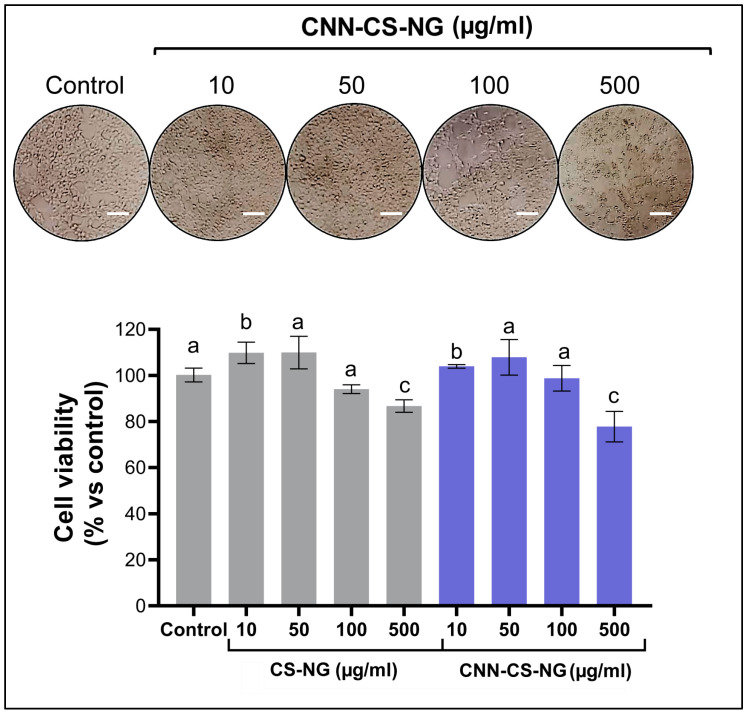
Phase-contrast microscopy images (scale bar: 50µm) and MTT analysis corresponding to neuronal SH-SY5Y cell line. Different letters indicate significant differences between experimental groups (*p* < 0.01).

Next, the in vitro cytocompatibility of both CS-NG and CNN-CS-NG was investigated in the system formed by human SH-SY5Y neuronal cell line using the metabolic viability through MTT assay. As shown in Figure 6, no significant reduction in cell viability was found when the NG concentration was equal to or less than 100 μg/mL up to 24 h of exposure. At the concentration of 500 μg/mL, cell viability was significantly reduced by 13.6% (CS-NG) and 22.2% (CNN-CS-NG). Also, cells suffered morphological changes since they lost cell–cell contact and rolled up rather than spreading out on the culture dish. Therefore, the obtained NG may not be considered harmful at a concentration less than 100 μg/mL for in vitro use.

**Figure 7:**
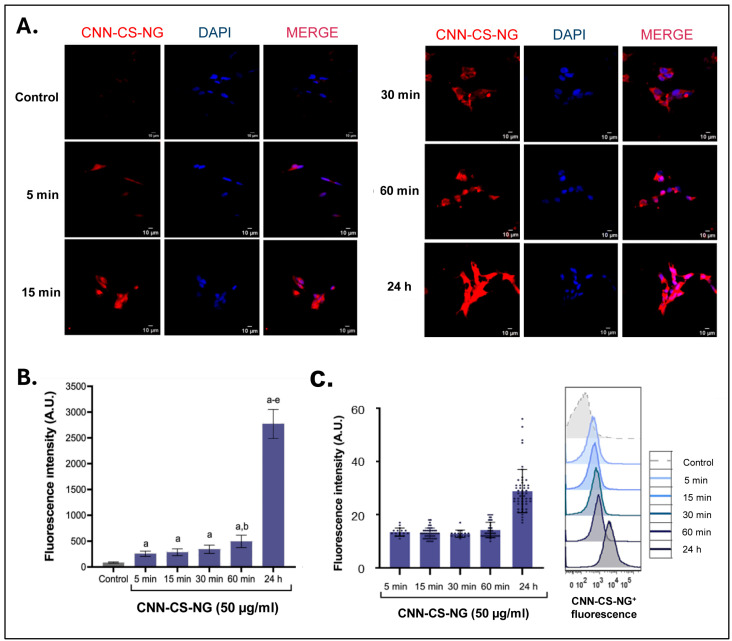
CLSM and flow cytometry analysis. (**A**) The CLSM images of neuronal SH-SY5Y cell line incubated with 50 µg/mL CNN-CS-NG for different times at 37 °C. Each series can be sorted by the nuclei of cells being dyed blue by DAPI, CNN NG, and a merge of the two channels of both above, respectively. Scale bar: 10 µm. (**B**) Mean fluorescence intensity bar-got plot. Dots represent the distribution of each quantify fluorescence intensity per cell determined from microscopy images. (**C**) Left: representative flow cytometer histograms showing the population of CNN-CS-NG-positive cells (blue scale) compared to the untreated control (gray). Right: quantification of the mean fluorescence intensity. Different letters indicate significant differences between experimental groups (*p* < 0.01).

In nanomedicine, the safe entry of NP into cells would be a crucial step to obtain high therapeutic efficacy when they are used as drug carriers. Therefore, the understanding of the uptake and the intracellular trafficking of NG is crucial for their design and development. Particularly, it is imperative to design carriers with the ability to escape from degradative vesicles. The latter phenomenon is called endo-lysosomal escape, and it has been well recognized as a major “bottleneck” in the drug delivery [33,43,44]. In the present report, the uptake capacity of the CNN-CS-NG (50 μg/mL) was studied as a function of time (0, 5, 15, 30, and 60 min and 24 h). To this end, representative images at each time were observed and captured with epifluorescence microscopy. Figure 7A shows that cells incorporated the NG gradually over time and they are widely internalized after 24 h of incubation. Images were taken maintaining the same setting parameters (brightness, contrast, exposure time). Then, changes in fluorescence intensity were quantified as a measure of particle uptake. Maximum fluorescence intensity was achieved at the highest time considered (Figure 7B). NG internalizations were confirmed by flow cytometry, which constitutes a kinetic approach. After CNN-CS-NG treatment, the resulting histogram of the fluorescent probe channel shifted to higher intensities in a time-dependent manner, resulting in a significant increase in fluorescence intensity into the cells. Particularly, the quantification determined that the mean values of fluorescence intensity (A.U.) for each recorded time was 340 (5 min), 345 (15 min), 418 (30 min), 483 (60 min), and 2641 (24 h) (Figure 7C). Both epifluorescence microscopy and cytometry techniques corroborated that the CNN-CS-NGs successfully entered the cells as a function of time.

**Figure 8:**
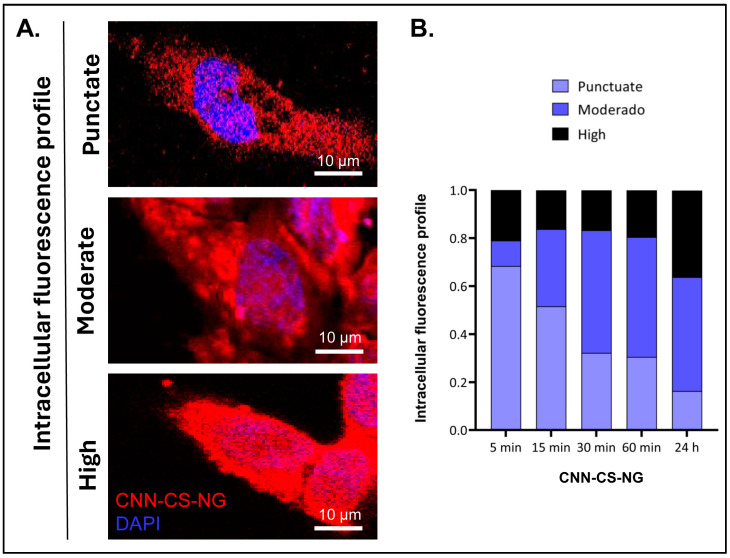
(**A**) IFP illustrating the difference between a punctate pattern (sequestered carrier into vesicles) and moderate/diffuse staining (cytosolic carrier). Scale bar: 10 µm. (**B**) Classification and counting of each pattern (*n* = 190 cells/experimental group).

Escape from endosomes/lysosomes was determined according to the ranking criteria previously described in Section 2.6.3 (item a). A punctate fluorescence pattern is frequently considered a sign of the tracer compound being entrapped into the vesicles, while a diffuse cytosolic staining implies leakage from them [32,43]. Three IFPs were detected and represented in Figure 8A: *punctate*, *moderate*, and *high*. Upon quantification, moderate and high IFP was mostly observed in cells after 24 h of CNN-CS-NP incubation in comparison to cells exposed for shorter time. Also, as the analysis time elapsed, it was feasible to detect the maturation of the endosomes and, finally, the escape into the cytoplasm. This is the reason why it was concluded that, at early incubation times, NGs are endocytosed. Then, the cationic nature of the present nanosystem would lead to the proton sponge effect, resulting in extensive osmotic swelling and eventual physical rupture of the acidic vesicles. 

**Figure 9:**
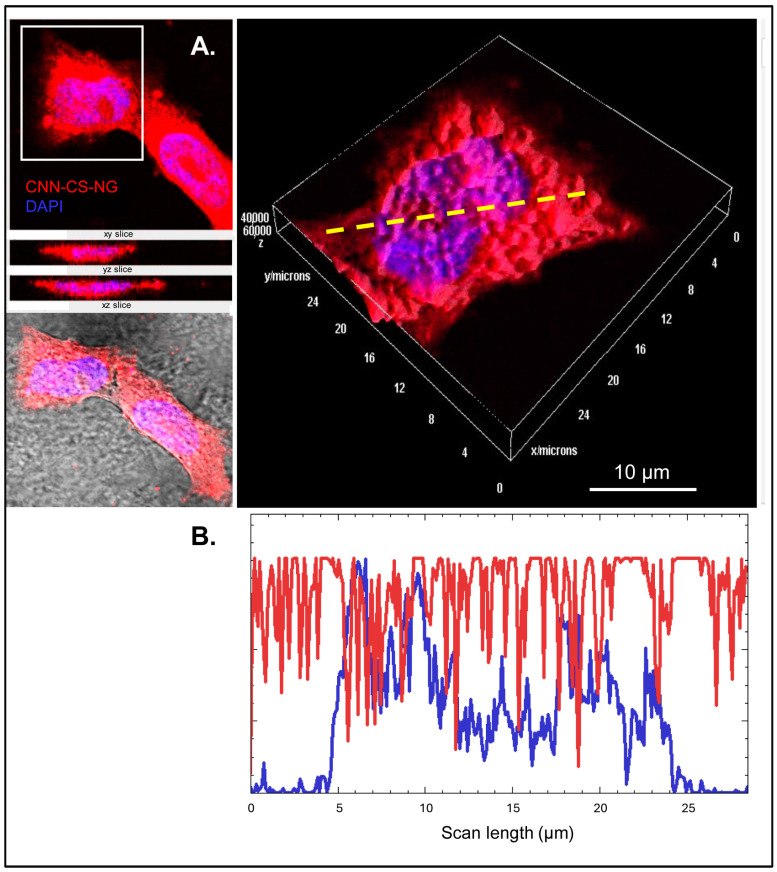
Intracellular localization of CNN-CS-NG in SH-SY5Y cells. (**A**) CLSM images of cells incubated 24 h with CNN-CS-NG (50 µg/mL) and stained by DAPI. (**B**) Line-scan profile fluorescence intensity perform through yellow dotted line. Scale bar: 10 μm.

Vermeulen et al. [45] observed that NGs accumulate in the nucleus upon endo-lysosomal release. In line with these authors, we observed that SH-SY5Y cells exhibited red fluorescence into the cytosol and nucleus upon 24 h of incubation (Figure 9A and Supplementary Video S1). This observation was corroborated by drawing a line-scan profile of fluorescent intensity with Fiji software (Figure 9B), which shows the red and blue signals coincident, suggesting the efficient nuclear uptake [46]. This can lead to the interpretation that CNN-CS-NGs were spread all over the cytoplasm, with a high amount in the nuclei.

### 3.4. NG Reach the Mice Brains

**Figure 10:**
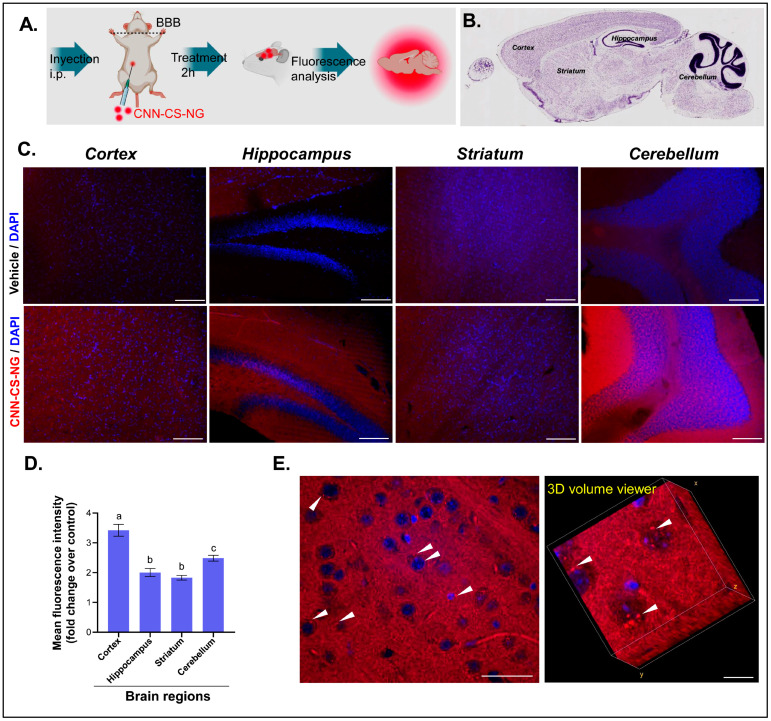
Images of different zones of mice brain. (**A**) Simplified representation of the experimental protocol. (**B**) Nissl stained-sagittal section obtained from Allen Institute for Brain Science, Atlas Brain Map to show the brain regions analyzed. (**C**) Microscopic brain images of both control (vehicle) and 1000 µg/mL CNN-CS-NG-treated mice (magnification of 10×, Scale bar 100 µm). (**D**) Quantification of the mean fluorescence intensities (fold change in control; bars show the mean ± SEM). Different letters indicate a statistically significant difference (*p* < 0.01). (**E**) Left, confocal image showing CNN-CS-NG as red spots inside the nervous cells (probably neurons) indicated with arrowheads (magnification of 60×, scale bar: 50 µm); right, 3D volume viewer (Fiji, plug in). Scale bar: 10 µm.

The ability of the CNN-CS-NG to reach CNS tissues was evaluated in vivo in healthy mice. A diagram of the protocol followed can be seen in Figure 10A. It was administered i.p. two concentrations of CNN-CS-NG (250 and 1000 μg/mL) and allowed to circulate for 2 h. The NG vehicle, distilled water, was used as a control (Figure 10A). Then, representative regions (cortex, striatum, hippocampus, and cerebellum) of the mouse brains were selected to examine whether CNN-CS-NG could be detected in sagittal slices (Figure 10B).

Figure 10C shows images taken with fluorescence microscopy of the abovementioned brain sections after the administration of 1000 μg/mL, compared to the corresponding control mouse brain. The results indicated that the mean fluorescence intensity was higher in brain regions that correspond to those treated in animals with 1000 μg/mL CNN-CS-NG. Meanwhile, no obvious differences were observed between the control and the 250 ug/mL CNN-CS-NG-treated animal group (Supplementary Figure S4). The results indicated a 3.2-fold increase in the cortex fluorescence, 2.5-fold in the cerebellum, and 2-fold in the striatal and hippocampal regions (Figure 10D). Remarkably, small perinuclear fluorescent spots were observed in samples after 2 h of administration (Figure 10E).

## 4. Discussion

One of the most important concerns when developing nanosystems for biomedical purposes is their ability to overcome biological barriers (e.g., BBB) and enter the intracellular space. Depending on the microscopy technique used (phase contrast microscopy, fluorescence microscopy, or TEM), NGs can be made detectable by binding/loading appropriate dyes during the generation process or by labelling with specific histochemical stain after administration [47]. Developing tracking approaches for nanosystems with fluorescent probes is a main issue in nanomedicine and especially in targeted drug delivery systems [48]. Among them, CNN-based probes have optimal photophysical and photochemical properties due to their good biocompatibility and low toxicity in living systems, with a wide range of applications in biomedicine and biochemistry. Current studies revealed that the combination of the properties of CNN and NG lead to the generation of versatile model platforms that allowed for the study of entrancing and metabolization of the nanosystem. Particularly, CNN–polymeric NG systems possess potential as probes for several types of imaging techniques [27,49]. Therefore, in the present study, CNN-CS-NGs were designed via the ionic gelation of a CS-CNN biopolymer with the objective of identifying its potential to be used as a drug carrier and delivery agent for neurological treatments. 

Regarding the NG size range, the literature reports that nanocarriers that promoted drug deposition in brain tissue should vary between 50 and 200 nm [36]. However, these studies consider only dry and naked NGs, which are represented only by their core area and do not exist in biological fluids. In the latter case, NGs have a hydration layer and are shaped by proteins that collect on their surfaces, resulting in a corona that enhances the hydrodynamic diameter of the particles. Manimarán et al. [15] explained that, depending on the precise use and desired properties, the size range of NGs used for targeted delivery can vary; that is to say, NGs < 50 nm often have advantages such as improved colloidal stability, higher efficient encapsulation of therapeutic molecules, and easy cellular uptake. On the other hand, NGs with sizes between 50 and 200 nm exhibit a good compromise between stability, drug-loading capacity, and circulation time, while those larger than 200 nm can provide prolonged circulation and improved stability. To gain insight into the properties of drug delivery nanovehicles in brain endothelial cells, the intracellular trafficking of fixed-size nanoparticles across an in vitro BBB model has been investigated based on different NP surface modifications. For example, latex NPs with a diameter ≥ 500 nm were internalized by nonphagocytic B16 cells through caveolae, whereas the uptake of particles up to 200 nm in diameter occurred by clathrin-mediated endocytosis in immortalized human brain capillary endothelial cell cultures [50]. Furthermore, in another report, caveolar endocytosis, adsorptive-mediated endocytosis, and receptor-mediated endocytosis were endorsed using uncoated 500 nm particles, attachment of the cationic polymer PEI, and attachment of prion proteins, correspondingly. In short, what the examples reveal is that the intracellular route of nanoparticles would be dominated by the characteristic of the particle surface rather than by a given size [51]. Similarly, Lombardo et al. mentioned in their review that NG size seems to have little impact in a size range from 12 to 340 nm, into which the BBB crossing was possible [52]. According to our results, the particle size that exceeded 200 nm seems to be adequate in both the in vitro uptake and the in vivo BBB passage models: fluorescent NGs were sent into cells. Particularly, CNN-CS-NG aggregates were observed using TEM and confocal microscopy. Nevertheless, the morphologies of these individual NG kept similar spherical structures. Analogous results were observed with FITC-CS NG and RBITC-CS NG generated by ionic gelation with TPP [53]. 

It should be noted that our fluorescent NGs were used as a proof of concept to determine whether CS- and TPP-based NGs can be proposed for drug delivery to the brain via i.p. administration. As such, we were able to confirm the presence of fluorescence in different brain areas, thus indicating that the NGs crossed the BBB. Notably, NGs without a fluorescent probe were smaller, as detected by DLS. We do not rule out that such an increase in size of CNN-CS-NGs could be due to the presence of CNN groups, since the same effect was stated by Monge Fuentes et al. [36]. In that report, mice received an i.p. injection of Albumin/PLGA-based NP (Al-NP) (340 nm, PdI 0.4 and −27 mM) or aluminum chloride phthalocyanine (AlClPc)–NP (497 nm, PdI 0.6 and −29 mM). Noteworthy, a considerable increase of around 46% in mean particle size was observed for Al-NPs functionalized with AlClPc when compared to native AL-NPs. Despite the mentioned size increase, Al-NP/AlClPc had the capacity to reach the brain parenchyma. At this point, our hypothesis is that particles larger than 500 nm may not reach the brain, while the smaller ones present in the colloidal solution are more prone to do so. Nevertheless, returning to the report of Monge Fuentes et al. [36], the authors revealed the presence of red fluorescent points throughout the brain (hippocampus and striatum) stating that they were precisely the NP’s agglomerates that emit light at 680 nm. 

The ζ-potential of CNN-CS-NG was slightly lower than that of the dye-free NG due to the consumption of amino groups by dye labelling, a reaction that was confirmed by FTIR analysis. Notably, the ζ-potential becomes a key parameter for the molecules that must cross cell membranes and, particularly, the BBB. This is due to the cytoplasmic membrane of endothelial cells having negative charges at physiological pH due to the presence of mucopolysaccharides, glycolipids, flucoproteins, proteoglycans, sialic acids, and sulphates [54]. In this sense, the electrostatic interaction between the positively charged part of the CNN-CS-NG and the negatively charges on the cell membrane could facilitate their absorption and transport through the endothelial cells, thus favoring their arrival to the brain parenchyma. Furthermore, Seko et al. reviewed that when the ζ-potential is high enough, e.g., greater than ± 30 mV, NGs repel each other. This event allows us to reach a dispersed solution preventing agglomeration during NGs’ solution storage. Particularly, aggregation begins at ζ-potential values less than 5 mV [10]. At first, the tendency is to hypothesize that nanovehicles with a ζ-potential value would not be able to cross the BBB. Nevertheless, fluorescent PEG-coated polystyrene NGs with a ζ-potential less negative than −4 mV were able to diffuse consistently in fresh human brain cortex, fresh rat ex vivo, and mouse brain in vivo [55]. Papadia et al., employing nanoliposomes with a similar size (100–150 nm) but with different ζ-potentials, determined a significant difference in the uptake of liposomes with neutral and non-neutral surface charge in hCMEC/D3 brain endothelium. These authors found no substantial variation in the cellular uptake between liposomes with a ζ-potential variation between −2 and −16 mV. Also, the greatest nominees to overcome the BBB resulted in being the electrically neutral molecules [56]. In conclusion, it could be concluded that ζ-potential did not exert a guiding role in the internalization processes.

Biomaterials are considered raw materials of biological origin capable of treating, improving, or supplanting tissues, organs, or functions in biological systems. These compounds play an increasingly important role in modern health application systems. Its biological impact is fundamental to the international standard ISO 10993-5, which provides guidelines for the choice of appropriate evaluations, such as cell damage due to morphological changes, measurements of cell growth, and/or specific aspects of cellular metabolism. All these techniques correspond to a classic approach that continues to be internationally accepted today. Thus, cytotoxicity can be addressed from multiple responses at the cellular level, one of the most used being the tetrazolium salt assay, better known as MTT. In this way, the effect of the tested concentrations of both CS-NG and CNN-CS-NG was evaluated on the percentage of metabolically active cells after a 24 h incubation period. The choice of concentrations was based on previous studies carried out in our laboratory [57]. It is worth mentioning that, in the human lines Caco-2 and ARPE-19, a significant proliferation was determined in CS presence. This situation has not been observed in SH-SY5Y cultures. However, the percentage of cell viability obtained was ≥ 85%. There is scarce information of the CS NG’s effect obtained by ionic gelation on SH-SY5Y cells. Bhattamisra et al. determined that FITC-CS NGs at doses less than 10 μg/mL were not cytotoxic, using the MTT method [58]. Also, FITC-carboxymethyl-CS-polyamidoamine dendrimers (200 μg/mL) with spherical morphology were successfully uptake after 24 h without affecting the cell viability of primary cultures of neurons and glial cells [59]. Del Prado-Audelo et al. described that, although the NG material was biodegradable and biocompatible, cytotoxicity depends on its parameters, i.e., size, charge, surface chemistry, and shape, and the concentration used. The interactions with the biological environment ultimately determine their potential cytotoxicity. In their report, the authors determined that the NG based on Pluronic^®^ F68 was not cytotoxic in a wide range of concentrations after 24 h of treatment in SH-SY5Y cells. In this sense, authors attribute this safety effect to factors that keep resemblance to our system; that is, the high biocompatibility of the polymer, the size, the moderate ζ-potential, and the spherical shape of the NG. Furthermore, the spherical morphology has been described as less toxic and less reactive compared to other shapes, such as fibers or nanotubes [60].

Rapid internalization was observed in SH-SY5Y cells cultured with CNN-CS-NG. For a constant fluorescence intensity, cellular uptake was time dependent. Lysosomal escape is critical, as this phenomenon limits the efficient administration of drugs in the cytoplasm and was well described for PEI, which is widely used as a vehicle to transfect cDNAs. Since the PEI molecule has numerous protonable amino groups, like CS, it exerts a pH buffer effect (proton sponge effect) that facilitates the escape of late endosomes or lysosomes, whose physiological pH is pH 5.5. When the accumulation of positive charges inside the vesicles is counteracted by a passive entry of chlorine ions to maintain neutrality, this large increase in ion concentration is accompanied by the entry of water molecules that cause vesicle swelling and the rupture of its membrane, consequently leading to the release of PEI NG and plasmids into the cytoplasm [33,43]. Similarly, in our previous report, it was proved that cationic polymeric NGs have pH buffering capacity and facilitate the lysosomal escape in ARPE-19 cells [23]. Here, fluorescence microscopy has the advantage of providing additional information on the intracellular fluorescence profile. A punctate fluorescence pattern is often considered an indication of the tracer. The compound is trapped in endosomes, while a diffuse label in the cytoplasm implies leakage of endosomal vesicles. However, it should be noted that this tracer cannot distinguish between endosomal escape by formation of pores in the lysosomal membrane or bursting. Yet, it can give an indication that the additional findings concerning intracellular trafficking events denoted the preferential nuclear localization and the escape of CNN NG from degradative lysosomal pathways [58,60]. 

In previous reports, animals were used to detect nanosystems in the brain. The presence of red or green fluorescence was analyzed depending on the probe used. DAPI was used to label the cell nuclei in certain works, such as this in report. In reference to this assay, it is important to highlight that the taken images showed red fluorescent areas not only in the tissue of the mice that received CNN-CS-NG but also in the control animals. As Khalin et al. also indicated, the control tissue has autofluorescence, that is, fluorescence not generated by the NG, but by the brain structures themselves, e.g., lipofuscin accumulation [25]. In any case, after correcting the levels of the positive signal above the autofluorescence signal, it is evident that the CNN-CS-NGs were able to reach the brain since the fluorescence intensity increased between 2 and 3 times. In a similar way, CS-TPP-based NGs functionalized with PEG and loaded with FITC were administered i.p. in mice to define their ability to cross the BBB and to reach the CNS [61]. Among the analyzed areas (hippocampus, cortex, striatum, corpus callosum, and thalamus), a predominant accumulation of NG in the hippocampus stands out in this report. Also, in accordance with our findings, FITC-CS NGs functionalized with PEG were detected closely in contact with nuclear structures, as shown by DAPI staining and inside neuronal cytoplasm, thus indicating, in this way, their capability in being endocited by cells. On the other hand, green fluorescent protein-CS nanosystems generated via a complex coacervation method could efficiently cross the BBB. However, the authors did not specify which brain area was analyzed; beyond this detail, their results suggested that green fluorescent protein–CS nanosystems could be efficient delivery vehicle for targeted therapies against brain cancers, between other brain diseases [17]. Fluorescent polystyrene NP with carboxylated- or polyethylene glycol-modified (PEGylated) surfaces were delivered into adult female mice. The exact anatomical distribution of the particles was examined by confocal microscopy after a short- and a long-time distribution period. The authors found NP signals with different fluorescent intensities in the brain, placenta, kidney, spleen, and liver after a single administration and displayed distinct clearing after 4 days. Interestingly, both types of NPs were detected in those detoxify organs. However, only organs protected by complex physiological barriers (brain and placenta) experimented the presence of carboxylated NP. A possible explanation lies in the fact that PEGylation reduced the attachment of particles to vessel walls [62].

Kaur et al. analyzed brain tissues excised from rats that received quantum dots (fluorescent probes)–PLGA-CS administered via the nasal route. Those particles were able to internalize in the brain after 30 min of administration, and an increasing red fluorescent intensity could be observed after 2 and 4 h of internalization in the left and right lobe, as well as in the cerebellum [63]. Albumin/PLGA nanosystems functionalized with photoactive compound aluminum chloride phthalocyanine were detected as agglomerates (fluorescent dots) at the striatum or hippocampal regions [36]. Also, brain sections exhibited rhodamine fluorescence corresponding to the presence of solid lipid NPs in the cerebral parenchyma. Particularly, red vesicles were detected inside the nervous cells and in the vasculature [54]. A series of derivatives of lipidized neurotransmitters, called NT-lipidoids, were doped into lipid NPs incapable of crossing the BBB. This doping allowed for the impermeability of the latter to be overcome, which was evidenced by the strong presence of tdTomato (fluorescent probe) signal in multiple regions of the brain, including the cerebral cortex, hippocampus, and cerebellum. In that report, the red fluorescence signal from hippocampal cells was weaker than that in cerebral cortex and cerebellum, indicating delivery efficiency up to this small region of the brain [64]. Khalin et al. [25] found that the red fluorescent signal present in the brain was 2-fold higher for rhodamine–PLGA NPs compared with PBS (vehicle). Finally, in a recent report, the presence of biodegradable poly(L-lactide) or non-biodegradable poly(perfluorodecyl acrylate)-based NPs in the brain of uninjured and brain-traumatized rats was evaluated. The fluorescent dye N-(2,6-diisopropylphenyl)-perylene-3,4-dicarboximide was used as a marker for the fluorescence measurements. Greater NP absorption was observed at 4 and 24 h after injection in the spleen and liver, followed by the kidney and brain, with minimal concentrations in the heart and lungs. The traumatized hemisphere showed greater fluorescent labelling, particularly in the perilesional area. To a lesser extent, signals were observed in areas far from the injury site and the contralateral hemisphere. The generated NG has the potential to serve as effective vehicles or markers for newly developed drugs with low or even no BBB permeation [65].

## 5. Conclusions

Scientific research from all around the world hopes to discover a cost-effective, simple, and minimally invasive method for beneficial bioactive compounds or drugs to cross the BBB. The BBB is not being targeted by many pharmaceutical companies, foundations, and funding agencies, thus making it more challenging to persuade them of the value of investing in this area. However, the positive results obtained by basic science, as shown in this report, will make it easier to move forward. Particularly, the cutting edge in nanotechnology generates optimism to overcome the growing challenges in biomedical sciences through the effective engineering of NGs. Of note, the novelty of this work is the interdisciplinary approach that encompasses chemistry, cellular biology, nanotechnology, and neurosciences at the expense of providing knowledge for the use of new pharmacological vehicles of accessible production even to lower- and upper-middle-income countries. In this way, a very simple, rapid, and inexpensive technique was used to carry out the proof of concept that NGs per se could cross the BBB, as well as being incorporated by cells of nervous origin and bypassing intracellular degradative pathways. At the same time, it was shown that the i.p. route of easy and agile application is as effective as the i.v. route, which requires greater technical complexity when manipulating rodents.

Among the pros of the encapsulation platform used here is the fact that we employed a biopolymer, the CS. This is a biomaterial with superior biocompatibility, as extensively stated in the Introduction section [66]. Concerning CS-based nanosystems, we list their lower cytotoxicity, their mucoadhesive character, their greater biocompatibility, and their stability. Furthermore, we believe that recurrent, expensive, and unpleasant doses could be avoided, and the therapeutic index of the drug could be increased. On the other hand, our experience tells us that the disadvantages could lie in the difficulty of controlling the colloidal stability of NG, i.e., the tendency of the particles to aggregate and even precipitate. Furthermore, the intrinsic properties of CS can be affected by the cross-linking preparation, and the method of NG generation must be adjusted according to the drug to be used and administered [67].

Overall, our findings demonstrate that NGs generated with CS and TPP, two reagents considered to be GRAS by the FDA of U.S. and European Medicines Agency (EMA), can be considered vehicles for neurological drugs or bioactive compounds.

## Data Availability

The data presented in this study are available upon request from the corresponding author. The data are not publicly available due to privacy.

## Supplementary Materials

The following supporting information can be downloaded at https://www.mdpi.com/article/10.3390/pharmaceutics16070964/s1. Figure S1: ^1^H NMR spectra of CS; Figure S2. (A) Lyophilized native and CNN-labelled CS. (B) Absorption spectra of CNN-CS (solid blue line) and CS (dashed black line) in H_2_O. (C) FTIR spectra for free CS molecule (black line) and CNN-CS (blue line). In the ATR-FTIR spectrum of CNN, broadening of the bands corresponding to the amines at 3268 nm (stretch of N-H bonds) is observed with respect to the unmodified CS, and the intensification and displacement of the characteristic absorption bands at 1645 cm^−1^ (C=O stretching of the amide II group) and 1582 cm^−1^ (N-H bending of primary amine) became gradually higher with the increase in amide bonds in the compound. Observable peak shifts with respect to native chitosan proved the successful conjugation of CNN to CS; Figure S3: MS ESI- spectra of CNN COOH. LC-MS quality supplies were used to prepare the solution. The sample was analyzed via direct injection in a high-resolution mass spectrometer with electrospray ionization source and quadrupole-time-of-flight analyzer model Xevo G2S Q-TOF (Waters Corp.). The ionization source parameters were optimized to maximize the signal-to-noise ratio, in ESI− and ESI+ modes; Figure S4: Microscopic brain images of 250 µg/mL CNN-CS-NG-treated mice (magnification of 10×, Scale bar 100 µm); Video S1: Cytoplasmatic and nuclear localization of CNN-CS-NG (red) in SH-SY5Y cells after 24 h of incubation. Images from all z-stacks are visualized through the rotation on the *y*-axis (Fiji software, version v1.54f). Nuclei were stained with DAPI (blue).

## Abbreviations

A.U., arbitrary units; BBB, blood–brain barrier; CLSM, confocal laser scanning microscopy; Cl-CNN, chlorotricarbocyanine; CNN, tricarbocyanine; CNN-CS-NG, tricarbocyanine–chitosan-based nanogels; CNS, central nervous system; CS, chitosan; CS-NG, chitosan based nanogels; D_2_O, deuterium oxide (heavy water); DD, degree of deacetylation; DAPI, 4′,6-diamidino-2-fenilindol; DCl, deuterium chloride solution; DMEM, Dulbecco’s Modified Eagle’s Medium; DMF, N,N-Dimethylformamide; DLS, dynamic light scattering; EDC, 1-ethyl-3-(3-dimethylaminopropyl) carbodiimide; FBS, foetal bovine serum; FTIR, fluorescein isothiocyanate; FTIR, Fourier-transform infrared spectroscopy; HIUS, high intensity ultrasound; i.p., intraperitoneal; IFP, intracellular fluorescence profile; NG, nanogels; NMR, nuclear magnetic resonance; NP, nanoparticles; MeOH, methanol; MS-ESI (M-H), electrospray ionization mass spectrometry; MTT, 3-(4, 5-dimethylthiazol-2-yl)-2, 5-diphenyltetrazolium bromide; PdI, polydispersity index; PEI, polyethyleneimine; PFA, paraformaldehyde; PLGA, poly(lactic-co-glycolic) acid; SEM, standard error of mean; SH-SY5Y, neuroblastoma cell line; TEM, transmission electron microscopy; TPP, sodium tripolyphosphate.
