# Supplementary figures and images for: Chitosan-Tricarbocyanine-Based Nanogels Were Able to Cross the Blood–Brain Barrier Showing Its Potential as a Targeted Site Delivery Agent
Source: Pharmaceutics. 2024 Jul 21;16(7):964. doi: 10.3390/pharmaceutics16070964 (PMC11280413; doi:10.3390/pharmaceutics16070964)

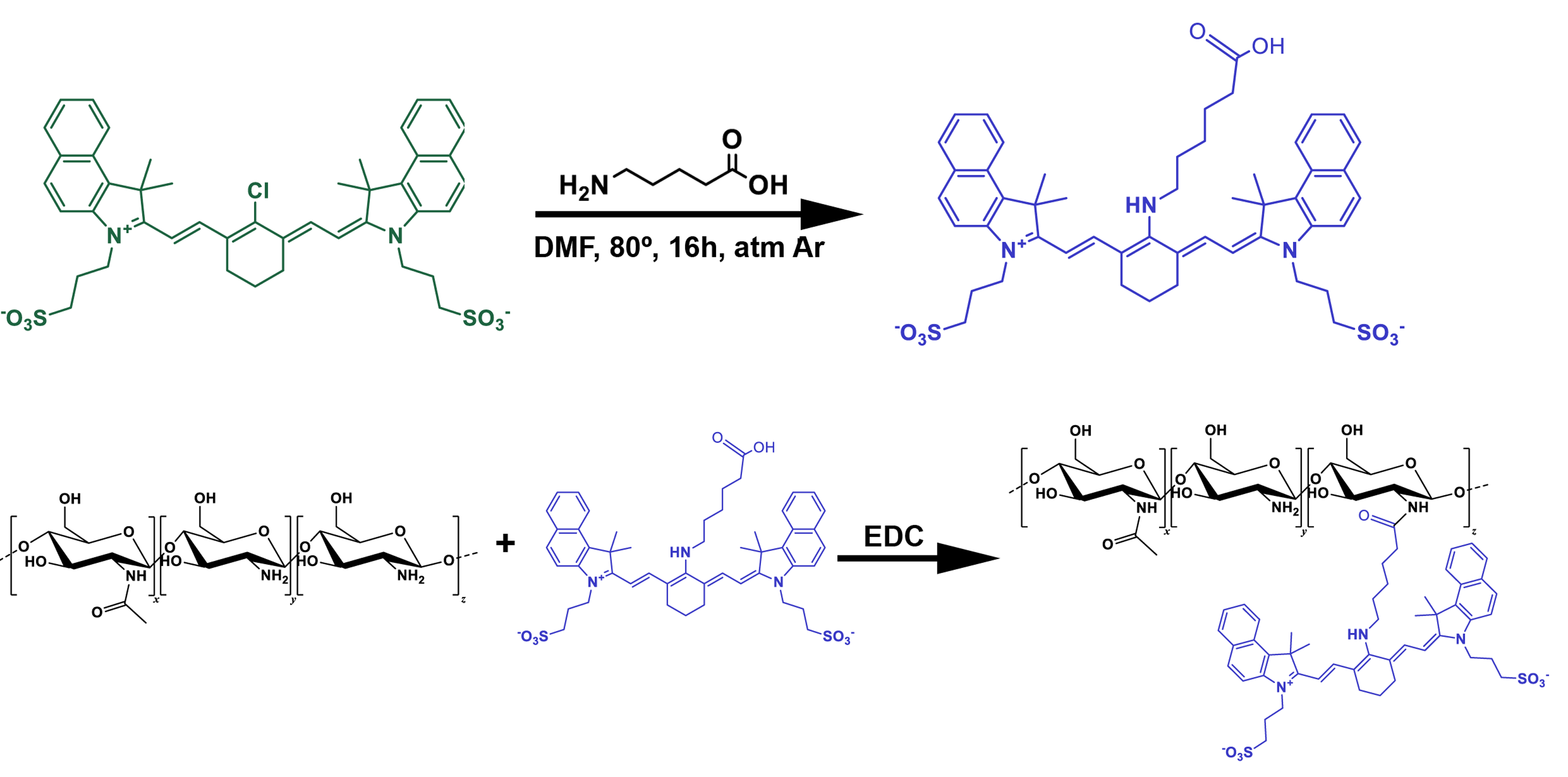

Supplement: Supplementary file 1 [file pharmaceutics-16-00964-s001.zip › Supplementary figures and video_final version/NEW Fig1.tif]

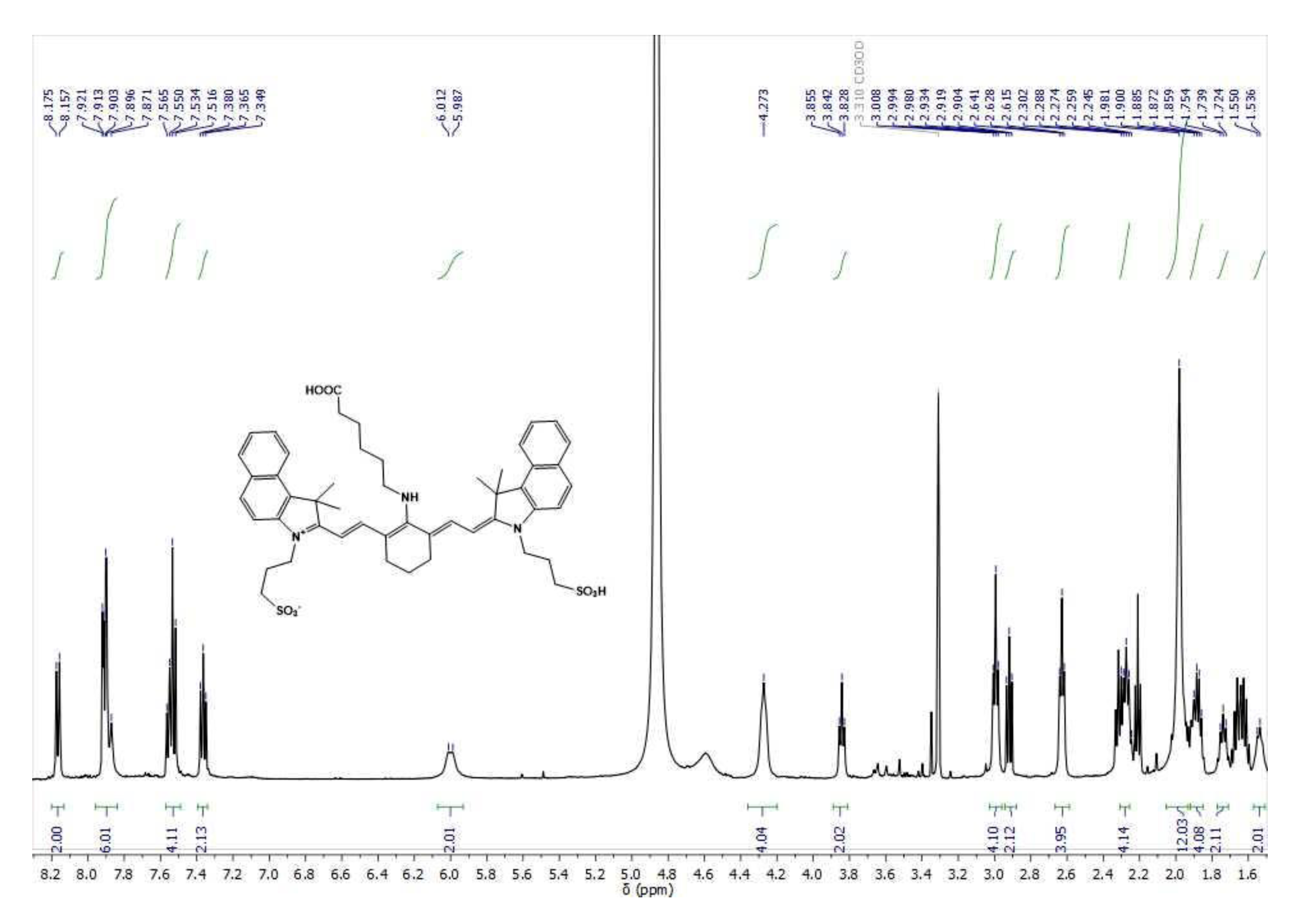

Supplement: Supplementary file 1 [file pharmaceutics-16-00964-s001.zip › Supplementary figures and video_final version/NEW Figure 1S.tif]

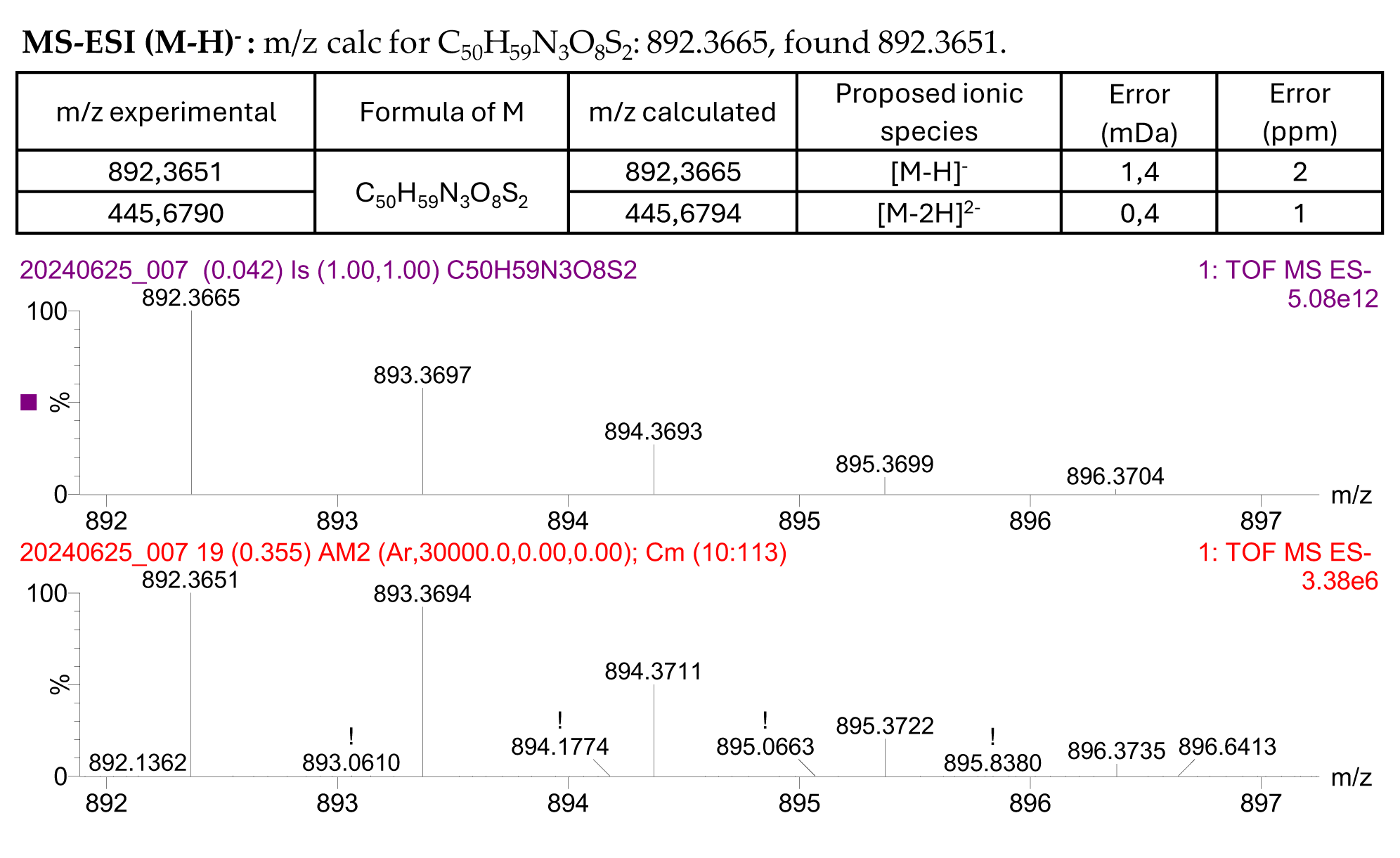

Supplement: Supplementary file 1 [file pharmaceutics-16-00964-s001.zip › Supplementary figures and video_final version/NEW Figure 3S.tif]

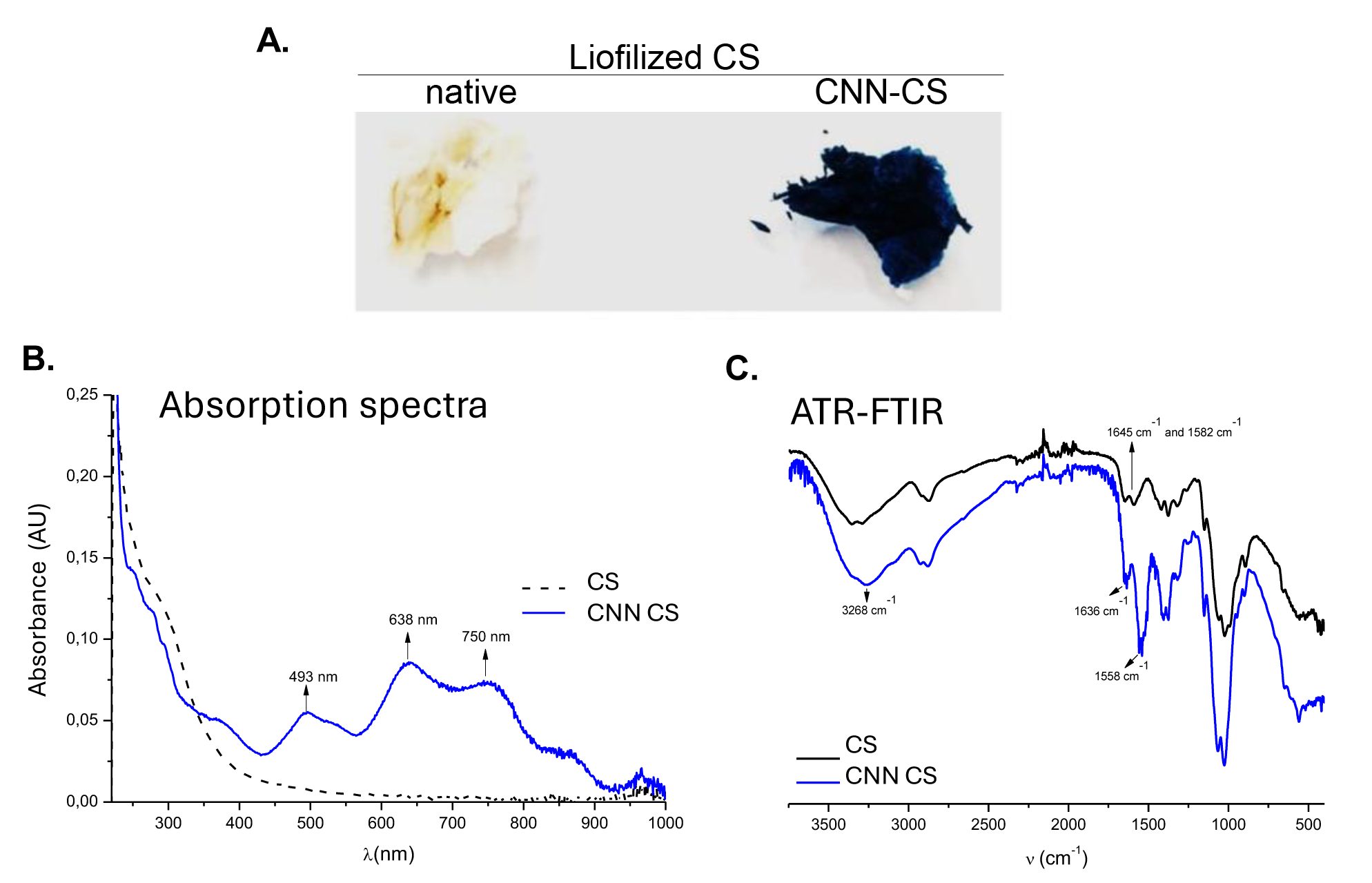

Supplement: Supplementary file 1 [file pharmaceutics-16-00964-s001.zip › Supplementary figures and video_final version/NEW Figure 2S.tif]

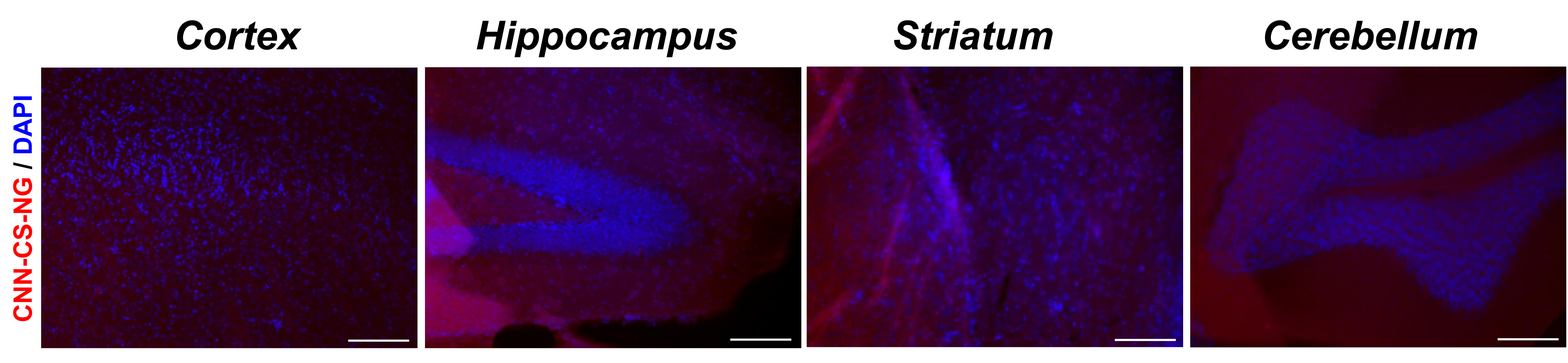

Supplement: Supplementary file 1 [file pharmaceutics-16-00964-s001.zip › Supplementary figures and video_final version/NEW Figure 4S.tif]
